# Minimal important differences for fatigue patient reported outcome measures—a systematic review

**DOI:** 10.1186/s12874-016-0167-6

**Published:** 2016-05-26

**Authors:** Åsa Nordin, Charles Taft, Åsa Lundgren-Nilsson, Anna Dencker

**Affiliations:** Gothenburg Centre for Person-Centred Care (GPCC), Sahlgrenska Academy, University of Gothenburg, Gothenburg, Sweden; Institute of Neuroscience and Physiology, Sahlgrenska Academy, University of Gothenburg, Gothenburg, Sweden; Institute of Health and Care Sciences, Sahlgrenska Academy, University of Gothenburg, Gothenburg, Sweden

**Keywords:** MID, Fatigue, PROM, Minimal important difference, Systematic review, MCID, MCII

## Abstract

**Background:**

Fatigue is the most frequent symptom reported by patients with chronic illnesses. As a subjective experience, fatigue is commonly assessed with patient-reported outcome measures (PROMs). Currently, there are more than 40 generic and disease-specific PROMs for assessing fatigue in use today. The interpretation of changes in PROM scores may be enhanced by estimates of the so-called minimal important difference (MID). MIDs are not fixed attributes of PROMs but rather vary in relation to estimation method, clinical and demographic characteristics of the study group, etc. The purpose of this paper is to compile published MIDs for fatigue PROMs, spanning diagnostic/patient groups and estimation methods, and to provide information relevant for appraising their appropriateness for use in specific clinical trials and in monitoring fatigue in defined patient groups in routine clinical practice.

**Methods:**

A systematic search of three databases (Scopus, CINAHL and Cochrane) for studies published between January 2000 to April 2015 using fatigue and variations of the term MID, e.g. MCID, MIC, etc. Two authors screened search hits and extracted data independently. Data regarding MIDs, anchors used and study designs were compiled in tables.

**Results:**

Included studies (*n =* 41) reported 60 studies or substudies estimating MID for 28 fatigue scales, subscales or single item measures in a variety of diagnostic groups and study designs. All studies used anchor-based methods, 21/60 measures also included distribution-based methods and 17/60 used triangulation of methods. Both similarities and dissimilarities were seen within the MIDs.

**Conclusions:**

Magnitudes of published MIDs for fatigue PROMs vary considerably. Information about the derivation of fatigue MIDs is needed to evaluate their applicability and suitability for use in clinical practice and research.

## Background

Fatigue is among the most frequent complaints reported by patients with chronic illnesses [1–4] and has far-ranging, often debilitating consequences on their wellbeing and physical, emotional and social functioning [5]. Although there is no consensus definition of fatigue, it is often described as ‘a persistent, overwhelming sense of tiredness, weakness or exhaustion resulting in a decreased capacity for physical and⁄or mental work [6]. Fatigue is a subjective experience and is commonly assessed by means of patient-reported outcome measures (PROMs). PROMs are widely used today in evaluating the effects of illness and treatment on symptoms, functioning, and other outcomes from the patient’s perspective [7].

Currently, there are some 40 generic and disease-specific PROMs for assessing fatigue in use today [8]. Most of these fatigue measures have been evaluated regarding various aspects of validity and reliability. Although these are important psychometric properties reflecting the quality of the measure, they are of little value in interpreting the meaning of scores derived from that measure [9]. Nonetheless, interpretation of scores, in particular changes in scores, is of critical concern in trials evaluating effects of treatments aimed at reducing fatigue, as well as in routine clinical practice in monitoring and managing fatigue in individual patients. In clinical trials, it has long been recognized that conventional statistical significance testing provides information regarding the probability that an effect exists, not about the meaningfulness of the size of the effect [10]. In clinical practice, difficulties in evaluating and interpreting changes in PROM scores often impinge on their usefulness in informing clinical decision-making [11].

The interpretation of changes in PROM scores may be enhanced by estimates of the so-called minimal important difference (MID). MID was originally defined over 25 years ago as “the smallest difference in score in the domain of interest which patients perceive as beneficial and which would mandate, in the absence of troublesome side effects and excessive cost, a change in the patient’s management” [12]. During the past decades considerable research attention has been directed towards deriving MIDs for PROMS. In this pursuit a variety of methods have been developed and applied, but no clear consensus exists regarding which method or methods are most suitable.

To date, two main methods have been applied, namely anchor-based approaches and distribution-based approaches. Descriptions of these methods are beyond the scope of this paper and are summarized in detail elsewhere [13]*.* Briefly, anchor-based approaches use various external criteria (patient-reported, physician-reported, or clinical anchors) to interpret whether a particular magnitude of change is important. For example, a common anchor-based method involves the use of global rating scales (GRS) where MIDs are derived by comparing patients’ self-ratings of change (e.g., “much worse”—“much better”) to change in PROM scores. The MID is often defined as lying within the range of “slightly worse/better” on the GRS [9]. Distribution-based approaches rely on the statistical characteristics of the distribution of scores in the sample, in which the magnitude of change is generally expressed as a function of the standard deviation (SD) of scores alone or in combination with the reliability of the PROM (standard error of the measurement (SEM)) [14]. Various SD and SEM cut-off values have been proposed for estimating MIDs, including ½ or 1/3 SD and 1–2 SEM. Another commonly applied method is the use of effect sizes (ES) or standardized response means (SRM), where change scores are divided by the SD at baseline or the SD of change, respectively. The MID is often defined as change values lying within the range of 0.2-0.5. A disadvantage to distribution-based approaches is that they do not address the clinical importance of the change. Recent recommendations have proposed that as a first-line method multiple anchor-based approaches should be used, which, supported by distribution-based methods, may be triangulated to a single MID value or smaller range of values [14–17].

Although appealing for its simplicity, the idea of a single, universal MID value for any particular PROM remains elusive for a number of reasons. Firstly, different MID estimation approaches have been shown to yield highly disparate MIDs and hence triangulation (combining different methods to estimate a MID) may be problematic [18]. Secondly, MIDs have also been shown to differ by population and context [14]. For example, MIDs vary by diagnostic group, characteristics of the study sample, e.g., demographics and baseline levels; disease severity; treatment; choice of anchors [18, 19] as well as if MIDs gauge improvement versus deterioration [20]. This variability suggests the need to understand how a particular MID value was determined in order to judge its appropriateness for use in research for interpreting change and/or computing sample sizes, or in clinical practice for monitoring fatigue in specific patient groups [21].

The purpose of this paper is to compile published MIDs for fatigue PROMs, spanning diagnostic/patient groups and estimation methods, and to provide information relevant for appraising their appropriateness for use in specific clinical trials and in monitoring fatigue in defined patient groups in routine clinical practice.

## Methods

A systematic literature review where three databases (Scopus, CINAHL and Cochrane) were searched from January 2000 to April 2015 to identify studies with calculated MIDs in fatigue scales, subscales and single item measures. The searches were limited to English language (search string: “minimal clinical important difference*” OR “minimal important difference*” OR “minimal clinically important difference*” OR “minimally important difference*” OR “clinical important improvement*” OR “clinically important improvement*” OR “minimal important clinical difference*” OR “minimally important clinical difference*” OR ”responder definition”) AND Fatigue). The search was augmented with screening of article reference lists. All expressions including “difference/change/improvement” or equivalent, “important” as well as “minimal” or “clinical”, or “responder definition” were defined as MIDs. To facilitate the reading all minimally important changes are called MIDs in this paper.

### Selection of articles

**Fig. 1:**
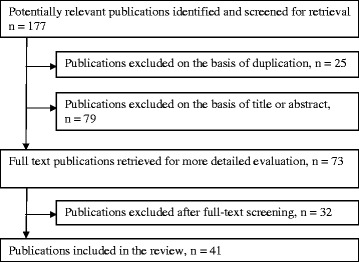
Flowchart of selection of articles to include

Inclusion criteria were reporting MIDs in text and/or tables for a fatigue scale, subscale or single item measurement of fatigue. Exclusion criteria were: reported MID was not derived directly in the study; insufficient information supplied about the study sample, study design and/or method for determining the MID; study sample < 18 years, not separate reporting of MIDs for a fatigue subscale and conference abstracts. Exclusion on title/abstract and on full-text levels were done independently by two researchers (ÅN and AD), see Fig. 1.

### Data extraction

Two authors (ÅN and AD) extracted data regarding MIDs and methods used, including anchors used. The last author (AD) checked all data extraction and prepared the tables. To facilitate interpretation all MIDs are shown as absolute values and decimals are restricted to one significant number only, except for effect sizes. Some studies reported standard deviation (SD) and confidence intervals but these are not shown in our tables or text. The fatigue measurements were identified as multidimensional scales, unidimensional scales or subscales, single item measurement or item bank scales.

## Results

The literature search generated 177 articles (Fig. 1), of which 41 met the inclusion criteria [22–62]. The main reasons for exclusion were: reported MID was not derived in the study; and inadequate information was supplied about the study sample, study design and/or method for determining the MID. Many different expressions were used to name a small but important change in fatigue [13]. In this review we included studies using different phrases for MID (see Table 1), e.g. “MID”, “MCID”, “MCII” or an equivalent expression, all referred to as MID in this paper. Most of these expressions used some variation of “difference/change/improvement” or equivalent, “important” as well as “minimal”. Some phrases also included “clinical”. Two studies used “responder definition” [43, 55], see Table 1. In two systematic reviews a phrase without “minimal” was used [59, 60] but the authors defined values for a small or minimal change.

**Table 1:** Included articles with reported fatigue MIDs, *n =* 41

First author/country	Instrument/scale	Term used^a^
Baró et al. 2011 [22] Spain	Perform Questionnaire	Minimally important difference, MID
Bedard et al. 2013a [23] Canada	ESAS fatigue item	Minimal clinically important difference, MCID
Bedard et al. 2013b [24] Canada	EORTC QLQ-30—Fatigue Scale	Minimal important difference, MID
Bedard et al. 2014 [25] Canada	EORTC QLQ-30—Fatigue Scale	Minimal important difference, MID
Bjorner et al. 2007 [26] USA, MOS-study	VT/SF-36	Minimally important difference, MID
Borghs et al. 2012 [27] Belgium	QOLIE-3 Energy/fatigue subscale	Minimally important change, MIC
Cella et al. 2002 [28] USA	FACIT-Fatigue TOI-F	Minimal clinically important difference, CID (MCID in short title)
Cella et al. 2005 [29] USA	FACT-Fatigue	Minimally important difference, MID
Colangelo et al. 2009 [30] Canada	Fatigue VAS (0–100)	Minimally important difference, MID
de Kleijn et al. 2011 [31] Netherlands	FAS	Minimal (clinically) important difference, MCID
George & Pope 2011 [32] Canada	VAS fatigue (0–100)	Minimal important difference, MID
Goligher et al. 2008 [33] Canada	MFI, FSS, MAF, CFS, FACIT-F, VT/SF-36, GRS	Minimal important difference, MID
Khanna et al. 2008 [34] Canada	Fatigue VAS (0–10)	Minimally important difference, MID
Kosinski et al. 2000 [35] USA	VT/SF-36	Minimally important change, MIC
Kvam et al. 2010 [36] Norway	EORTC QLQ-C30 Fatigue Subscale	Minimal important difference, MID
Kwok and Pope 2010 [37] Canada	Fatigue VAS (0–100)	Minimally important difference, MID
Lai et al. 2011 [38] USA	FACIT-Fatigue subscale	Minimally important difference, MID
Lasch et al. 2009 [39] USA	SIS Energy/Fatigue and Mental Fatigue subscales	Minimum important difference, MID
Maringwa et al. 2011a [40] 17 countries	EORTC QLQ-C30 Fatigue Subscale	Minimal clinically important difference, MCID
Maringwa et al. 2011b [41] 12 countries	EORTC QLQ-C30 Fatigue Subscale	Minimal important difference, MID
Mathias et al. 2009 [42] USA and Europe	ITP-PAC	Minimally important difference, MID
Matza et al. 2013 [43] USA	FAsD	Responder definition
Mills et al. 2012 [44] UK	NFI-MS	Minimum clinically important difference, MCID
Patrick et al. 2003 [45] USA	FACT-An fatigue subscale	Minimally important difference, MID
Pouchot et al. 2008 [46] Canada	MFI, FSS, MAF, CFS, FACIT-F, VT/SF-36, GRS	Minimal clinically important difference, MCID
Purcell et al. 2010 [47] Australia	MFI subscales	Minimal clinically importantdifference, MCID
Reddy et al. 2007 [48] USA	FACIT-Fatigue subscale ESAS fatigue item	Clinically important improvement
Rendas-Baum et al. 2010 [49] Canada	FIS	Minimally important difference, MID
Robinson et al. 2009 [50] USA	FSS	Minimally important difference, MID
Schwartz et al. 2002 [51] USA	SCFS, POMS-F, single item	Minimally important clinical difference, MICD
Schünemann et al. 2005 [52]	CRQ/Fatigue subscale	Minimal important difference, MID
Sekhon et al. 2010 [53] Canada	Fatigue VAS (0–100)	Minimally important difference, MID
Spiegel et al. 2005 [54]	VT/SF-36	Minimally clinically important difference, MCID
Twiss et al. 2010 [55] 8 countries	U-FIS	Responder definition, RD
Ward et al. 2015 [56] USA	VT/SF-36	Minimal clinically important improvement, MCII
Wells et al. 2007 [57] USA	Fatigue VAS (0–100)	Minimal clinically important difference, MCID
Wheaton & Pope 2010 [58] Canada	Fatigue VAS (0–100)	Minimal important difference, MID
Wyrwich et al. 2003 [59]	VT/SF-36 CRQ/Fatigue subscale	Clinically important difference, CID
Wyrwich et al. 2004 [60]	VT/SF-36 CHQ/Fatigue subscale	Clinically important difference, CID
Yost et al. 2011 [61] USA	PROMIS Fatigue (Fatigue-17, Fatigue-7)	Minimally important difference, MID
Zeng et al. 2012 [62] 7 countries	EORTC QLQ-C30 Fatigue Subscale	Minimal clinically important difference, MCID

^a^All expressions in this column are referred to as “MID” in the current study

The included articles (*n =* 41) reported MIDs for 28 fatigue PROMs (characteristics shown in Table 2), resulting in 60 studies/substudies of MIDs. The studies varied in sample size, diagnostic group, MID estimation approach, study design, type of intervention and length of follow up. Sample sizes ranged from *n =* 40 to *n =* 2,583. Sixteen different diagnoses were included in the reviewed studies. Twenty-seven of the studies in the 41 articles were longitudinal and follow-up periods ranged from two days after intervention to one year after baseline. An anchor-based approach alone was used in 39 of the 60 studies or substudies estimating MID, while the rest also used a distribution-based approach. Seventeen of these also included a method of triangulation to define MIDs. Two cross-sectional studies [33, 46] reported MIDs for seven fatigue or vitality scales (MFI, FSS, MAF, CFS, VT/SF-36, FACIT-F and GRS). Other studies determined MIDs for two or more fatigue measures or subscales [28, 47, 48, 51, 59–61]. Several PROMs had MIDs determined in a number of different studies and several studies reported MIDs for up to seven PROMs. Nevertheless, most MIDs were derived in single studies, with one study per PROM [22–27, 29–32, 34–43, 45, 49, 50, 52–58, 62], see Table 3. Altogether, 60 studies or substudies estimating MIDs for global change (not specified direction of change), improvement and/or deterioration are described in Table 3. In Table 3 all score changes are presented as positive values, regardless of the direction of change. Confidence intervals and SDs (if derived in study) are not shown. Numbers are rounded to one decimal place.

**Table 2:** Overview of reviewed fatigue scales, subscales and single fatigue item with published MIDs, *n =* 28

Name of PROM	Abbreviated name	Number of items	Score range	Characteristics of scale
Chalder Fatigue Scale	CFS	14	0–33	Multidimensional
Chronic Heart Failure Questionnaire, Fatigue subscale	CHQ	4	1–7	Unidimensional subscale Negative score^a^
Chronic Respiratory Questionnaire, Fatigue subscale	CRQ	4	1–7	Unidimensional subscale Negative score^a^
Edmonton Symptom Assessment System, Fatigue item	ESAS Fatigue item	1	0–10	Single item
European Organization for Research and Treatment of Cancer Quality of Life Questionnaire Core 30, Fatigue Subscale	EORTC QLQ-30 Fatigue Subscale	3	0–100	Unidimensional subscale
FACIT-Fatigue Scale	FACIT-Fatigue	13	0–52	Unidimensional scale/subscale, Negative score^a^
FACT-An, Fatigue subscale	FACT-An Fatigue	20	0–80	Unidimensional subscale
Fatigue Assessment Scale	FAS	10	10–50	Unidimensional
Fatigue Associated with Depression Questionnaire	FAsD	13	1–5	Multidimensional
Fatigue Impact Scale	FIS	40	0–160	Multidimensional
Fatigue Severity Scale	FSS	9	1–7	Multidimensional
Global RS	GRS	1	0–10	Single item
Immune thrombocytopenic Purpura –Patient Assessment Questionnaire, Fatigue subscale	ITP-PAC	1	0–100	Single item
Multidimensional Assessment of Fatigue	MAF	16	1–50	Multidimensional
Multidimensional Fatigue Inventory	MFI	20	20–100 (4–20 in each subscale)	Multidimensional, (5 subscales: GF, PF, RA, RM and MF)^b^
Neurological Fatigue Index for multiple sclerosis	NFI-MS	12	0–30 in SS, 0–24 in PS and 0–12 in CS	Multidimensional, (3 scales: SS, PS and CS)^c^
Perform Questionnaire	PQ	12	12–60	Multidimensional, Negative score^a^
Profile of Mood States-Fatigue	POMS-F	7	0–28	Unidimensional subscale
PROMIS Fatigue-17	Fatigue-17	17	17–85	Unidimensional
PROMIS Fatigue-7	Fatigue-7	7	7–35	Unidimensional
Quality of Life Inventory in Epilepsy, Energy/Fatigue subscale	QOLIE-31	4	0–100	Unidimensional subscale Negative score^a^
Schwartz Cancer Fatigue Scale	SCFS	6	6–30	Multidimensional
SF-36 Vitality scale	SF-VT	4	0–100	Unidimensional subscale
Sleep Impact Scale, Energy/Fatigue (E/F), Mental Fatigue (MF) subscales	SIS	5 (E/F) 3 (MF)	0–100	Unidimensional subscales Negative scores^a^
Trial Outcome Index-Fatigue	TOI-F	27	0–108	Multidimensional, Negative score^a^
Unidimensional Fatigue Impact Scale	U-FIS	22	0–66	Unidimensional
Visual Analogue Scale 0–10	VAS 0–10	1	0–10	Single item
Visual Analogue Scale 0–100	VAS 0–100	1	0–100	Single item

^a^Negative score = high values indicate low level of fatigue

^b^ *GF* General fatigue, *PF* Physical fatigue, *RA* Reduced activity, *RM* Reduced motivation. *MF* Mental fatigue

^c^ *SS* Summary scale, *PS* Physical scale and *CS* Cognitive scale

**Table 3:** Studies/substudies (*n =* 60) with reported MIDs for fatigue scales, subscales or single fatigue items. Study design and population are shown along with estimation method

Instrument/scale (score range)	Citation/Population	Design	Anchor-based MID				Distribution-based MID	Triangulation MID
			Anchor	Global change (% of scale range)	Improved	Worsened		
Multidimensional scales								
MFI (20–100)	Goligher et al. 2008 SLE (*n =* 80)	Cross-sectional	GRS (7-step) Paired comparisons	11.5 (14 %)	9.6	12.8		
	Pouchot et al. 2008 RA (*n =* 61)	Cross-sectional	GRS (7-step) Paired comparisons	13.3 (17 %)	6.8	9.5		
FSS (1–7)	Goligher et al. 2008 SLE (*n =* 80)	Cross-sectional	GRS (7-step) Paired comparisons	0.6 (10 %)	0.08	1.2		
	Pouchot et al. 2008 RA (*n =* 61)	Cross-sectional	GRS (7-step) Paired comparisons	1.2 (20 %)	0.4	1.0		
	Robinson et al. 2009 MS (*n =* 249)	Cross-sectional	Disease duration, Expanded Disability Status Scale, Patient Assessment of MS Impact, MS Functional Composite	0.5–1.1 (8–18 %)			ES 0.3–0.8	1
MAF (1–50)	Goligher et al. 2008 SLE (*n =* 80)	Cross-sectional	GRS (7-step) Paired comparisons	5.0 (10 %)	1.4	8.9		
	Pouchot et al. 2008 RA (*n =* 61)	Cross-sectional	GRS (7-step) Paired comparisons	9.2 (19 %)	5.4	8.3		
CFS (0–33)	Goligher et al. 2008 SLE (*n =* 80)	Cross-sectional	GRS (7-step) Paired comparisons	2.3 (7 %)	0.7	3.2		
	Pouchot et al. 2008 RA (*n =* 61)	Cross-sectional	GRS (7-step) Paired comparisons	3.3 (10 %)	1.4	3.5		
FIS (0–160)	Rendas-Baum et al. 2010 MS (*n =* 184)	Cross-sectional	Expanded Disability Status Scale (EDSS), SF-36, EQ-5D	9–24 15.5 (10 %)			4.8 (1 SEM), 9.6 (2 SEM) 11.6 (1/3 SD), 17.3 (½ SD)	10–20
TOI-F (0–108)	Cella et al. 2002 Cancer (*n =* 2,583)	Cross-sectional Longitudinal 3 studies, follow-up: 3 d - 12 m	Performance status, haemoglobin level, response to treatment	4.8–26.6 (4–25 %)			4.2 (1 SEM), 10.5 (½SD)	5.0
PQ (12–60)	Baró et al. 2011 Cancer (*n =* 437)	Longitudinal Follow-up: 3 m	Haemoglobin level		3.7			3.5
SCFS (6–30)	Schwartz et al. 2001 Cancer (*n =* 103)	Longitudinal Follow-up: 2 d	GRS (7-step)	5.0 (21 %)	2.1	5.7		
FAsD (1–5)	Matza et al. 2013 Depression (*n =* 96)	Longitudinal Follow-up: 6 w	BFI, ESS, CGI-S, Patient’s perception of change		0.3–0.6	0.2–0.3		
NFI-MS (0–30, 0–24 and 0–12 resp.)	Mills et al. 2012 MS (*n =* 208)	Longitudinal Follow-up: 6–8 w	Global perceived change item (5-step)	2.5 (SS) (8 %) 2.4 (PS) (10 %) 0.8 (CS) (7 %)				
Unidimensional scales or subscales								
MFI sbscales: GF, PF, RA, RM and MF (4–20)	Purcell et al. 2010 Cancer (*n =* 210)	Longitudinal Follow-up: 6 w post treatment	Score change pre- and post-radiotherapy	GF: 2.1 (13 %) PF: 2.0 (13 %) RA: 2.4 (15 %) RM: 1.6 (10 %) MF: 1.4 (9 %)				2 for each subscale
U-FIS (0–66)	Twiss et al. 2010 MS (*n =* 911)	Longitudinal Follow-up: 12 m	EQ-5D		6.5	4.7	4.2–7.0 (ES 0.3–0.5) 2.4 (1 SEM)	
FAS (10–50)	de Kleijn et al. 2011 Sarcoidosis (*n =* 321)	Longitudinal Follow-up: 12 m	WHOQOL-BREF/Physical health domain, ROC	3.5 (9 %)	3.0	3.8	4.2 (ES 0.5) 3.6 (1 SEM)	4
SF-36 VT (0–100)	Bjorner et al. 2007 Several disease conditions (*n =* 3,445)	Cross-sectional	Regression analyses using age, gender, race, disease condition and functional outcomes					5/group level 10/individual level
	Goligher et al. 2008 SLE (*n =* 80)	Cross-sectional	GRS (7-step) Paired comparisons	10.7 (11 %)	7.3	18.3		
	Kosinski et al. 2000 RA (*n =* 693)	Longitudinal Follow-up: 6 w	Patient global assessment, Physician global assessment, pain, swelling, tenderness	4.9–11.1 (5–11 %)				
	Pouchot et al. 2008 RA (*n =* 61)	Cross-sectional	GRS (7-step) Paired comparisons	14.8 (15 %)	11.3	11.9		
	Spiegel et al. 2005 Hepatitis C virus	Systematic review Delphi method	ES data from included studies				ES of 0.2 Range 0.15–0.25	4.2 Range 3–5
	Ward et al. 2015 RA (*n =* 249)	Longitudinal Follow-up: 1–4 m	HAQ, CES-D, Health transition item of SF36, Global transition item	11.0–20.0 (11–20 %)				
	Wyrwich et al. 2003 Chronic obstructive pulmonary disease	Delphi method	Patient change scenarios and SF36 data					12.5
	Wyrwich et al. 2004 Coronary artery disease/congestive heart failure	Delphi method	Patient change scenarios and SF36 data					18.8 Range 18.8–25
FACIT-Fatigue (0–52)	Cella et al. 2002 Cancer (*n =* 2,583)	Cross-sectional Longitudinal 3 d - 12 m	Performance status, haemoglobin level, response to treatment	3 (6 %)				3
	Cella et al. 2005 RA (*n =* 271)	Longitudinal Follow-up: 24 w	VT/SF-36, MAF	3–4 (6–8 %)			4.10 (1 SEM) 2.2–5.5 (ES 0.2–0.5)	3–4
	Goligher et al. 2008 SLE (*n =* 80)	Cross-sectional	GRS (7-step) Paired comparisons	5.9 (11 %)	2.8	9.1		
	Lai et al. 2011 SLE (*n =* 254)	Longitudinal Follow-up: 12–52 w	Physician-reported anchors (Physician GA)	3–7 (6–13 %)			2.7 (1 SEM) 4.6 (1/3 SD), 6.8 (½SD)	3–6
	Pouchot et al. 2008 RA (*n =* 61)	Cross-sectional	GRS (7-step) Paired comparisons	8.3 (16 %)	6.8	5.2		
	Reddy et al. 2007 Cancer (*n =* 194)	Longitudinal Follow-up: 8 d	Global Benefit Score (7 step)		10			
FACT-An Fatigue (0–80)	Patrick et al. 2003 Cancer (*n =* 375)	Longitudinal, Follow-up: pre and post chemotherapy	Haemoglobin level Regression analysis		4.2			
POMS-F (0–28)	Schwartz et al. 2002 Cancer (*n =* 103)	Longitudinal Follow-up: 2 d	GRS (7-step)	5.6 (20 %)	2.1	5.7		
EORTC QLQ-C30 Fatigue Subscale (0–100)	Bedard et al. 2013b Cancer (*n =* 276)	Longitudinal Follow-up: 1 m	Overall QoL			24.5	19.7 (1 SEM) 6–15 (0.2–0.5 SD)	
	Bedard et al. 2014 Cancer (*n =* 369)	Longitudinal Follow-up: 1 m	Overall QoL 1–7 Overall health anchor		13.6–17.3		1.8 (1 SEM) 6.7–16.8 (0.2–0.5 SD)	
	Kvam et al. 2010 Multiple myeloma (*n =* 239)	Longitudinal Follow-up: 3 m	Global rating of change (7-step but categorized into 3)		13.5	8.6		
	Maringwa et al. 2011a Brain cancer (*n =* 941)	Cross-sectional and longitudinal	WHO Performance Status and MMSE		12.4	8.9	10.0 (1 SEM)	
	Maringwa et al. 2011b Lung cancer (*n =* 812)	Cross-sectional and longitudinal	Physician-rated WHO PS and weight change		14.1	5.7	11 (1 SEM)	
	Zeng et al. 2012 Cancer (*n =* 93)	Longitudinal Follow-up: 1 m	KPS clinical marker		11.4	7.8	3.0–3.1 (1 SEM) 5.8–14–7 (0.2–0.5 SD)	
SIS (0–100)	Lasch et al. 2009 MDD (*n =* 379)	Longitudinal Follow-up: 8 w	Clinician rated tool (7-step) on severity and improvement	E/F: 11.9 (12 %) MF: 13.3 (13 %)			8.7 (½SD) 10.6 (½SD)	
CRQ (1–7)	Schünemann et al. 2005 Chronic obstructive pulmonary disease	Systematic review	CRQ data from5 studies, patient global ratings anchors and distributions based MIDs	0.5–0.6 (8–10 %)			0.47–0.54 (1 SEM)	0.5
	Wyrwich et al. 2003 Chronic obstructive pulmonary disease	Delphi method	Patient change scenarios and CHQ data					2
CHQ (4–28)	Wyrwich et al. 2004 Coronary artery disease/congestive heart failure	Delphi method	Patient change scenarios and CHQ data					3 Range 3–4
QOLIE-31 Energy/fatigue subscale (0–100)	Borghs et al. 2012 Epilepsy (*n =* 1,035)	Longitudinal study of 3 RCTs Follow-up: 12 w	Patient global impression of change (PGIC) Regression analysis	7.5 (8 %)			5.8 (0.3 ES) 9.4 (1 SEM)	
Single item measure								
VAS Fatigue Single item (0–100)	Colangelo et al. 2009 SLE (*n =* 202)	Longitudinal Follow-up: 7.5 m	Self-rated health (5 step)		13.9	9.1		
	George & Pope 2011 Sjögren’s syndrome (*n =* 40)	Longitudinal Follow-up: ≤ 16 m	Self-rated health (5 step)		6.2	15.2		
	Kwok & Pope 2010 PsA (*n =* 200)	Longitudinal Follow-up: ≤12 m	Self-rated health (5 step)		8.2	3.6		
	Sekhon et al. 2010 Systematic sclerosis (*n =* 109)	Longitudinal Follow-up: 7.5 m	Self-rated health (5 step)		10.0	3.8		
	Wells et al. 2007 RA (*n =* 1,043)	Longitudinal Follow-up: 6–12 m Delphi method	HAQ, Patient Global assessment of disease and pain	6.7–17 (7–17 %)				10
	Wheaton & Pope 2010 SpA (*n =* 140)	Longitudinal Follow-up: 5 m	Self-rated health (5 step)		1.4	14.4		
VAS Fatigue Single item (0–10)	Khanna et al. 2008 RA (*n =* 307)	Longitudinal Follow-up: 5.9 m	Retrospective anchor (5-step)		0.8–1.1	1.1–1.3	Improved: ES = 0.39 Worsened: ES = 0.44	
GRS Single item (0–10)	Goligher et al. 2008 SLE (*n =* 80)	Cross-sectional	GRS (7-step) Paired comparisons	1.3 (13 %)	0.3	1.5		
	Pouchot et al. 2008 RA (*n =* 61)	Cross-sectional	GRS (7-step) Paired comparisons	2.0 (20 %)	0.9	1.5		
	Schwartz et al. 2001 Cancer (*n =* 103)	Longitudinal Follow-up: 2 d	GRS (7-step)	1.1 (11 %)				
ESAS fatigue Single item (0–10)	Bedard et al. 2013a Cancer (*n =* 421)	Longitudinal Follow-up: 4–12 w	Well-being		0.1–1.3	1.0–1.8	0.1 (1 SEM) 0.5–1.4 (0.2–0.5 SD)	
	Reddy et al. 2007 Cancer (*n =* 194)	Longitudinal Follow-up: 8 d	Global Benefit Score (7 step)		4			
ITP-PAC (0–100)	Mathias et al. 2009 ITP (*n =* 125)	Longitudinal Follow-up: 4 w	Global assessment of change items (15-step)	15.0 (15 %)			ES = 0.57	
PROMIS fatigue item bank scales								
PROMIS Fatigue-17 (17–85)	Yost et al. 2011 Cancer (*n =* 101)	Cross-sectional and longitudinal Follow-up: 6–12 w	23 anchor measures	T-score MID: 2.5–4.5 Raw-score MID: 4.0–8.0 (6–12 %)			Cross-sectional: ES 0.34–0.79 Longitudinal: ES 0.27–0.52	
PROMIS Fatigue-7 (7–35)	Yost et al. 2011 Cancer (*n =* 101)	Cross-sectional and longitudinal Follow-up: 6–12 w	23 anchor measures	T-score MID: 3.0–5.0 Raw-score MID: 2.0–3.0 (7–11 %)			Cross-sectional: ES 0.24–0.76 Longitudinal: ES 0.24–0.51	

GRS (7-step) = Global rating scale with 7 response categories: Much more fatigue, Somewhat more fatigue, A little bit more fatigue, About the same fatigue, A little bit less fatigue, Somewhat less fatigue, and Much less fatigue

Paired comparisons = Participants rated their fatigue in relation to another participant

*PsA* Psoriatic arthritis, *SpA* Spondyloarthropathy

### Multidimensional scales

#### Multidimensional fatigue inventory (MFI), score 20–100

Two cross-sectional studies [33, 46] derived MIDs for systemic lupus erythematosus (SLE) and rheumatoid arthritis (RA) populations for the MFI total scale, using a patient global rating scale and interviews as anchors. MIDs ranged from 11.5 to 13.3 for global change and 6.8 to 9.6 for improvement and 9.5 and 12.8 for deterioration.

#### Fatigue severity scale (FSS), score 1–7

Three cross-sectional studies reporting MIDs for the FSS were identified [33, 46, 50]. Diagnostic groups included SLE, RA and multiple sclerosis (MS). Anchor-based approaches were applied in all the three studies and a distribution-based approach (viz. effect size, ES, of at least 0.25) was also applied in one [50]. Two used a patient global rating scale as an anchor [33, 46] whereas the third used clinical anchors and baseline data from a clinical trial to establish MIDs [50] MIDs ranged from 0.5 to 1.2 for global change, 0.08 to 0.4 for improvement and 1.0 to 1.2 for deterioration.

#### Multidimensional assessment of fatigue (MAF), score 1–50

MID-estimates for the MAF in two cross-sectional studies with SLE and RA patients [33, 46] were estimated to 5.0 and 9.2 for global change, 1.4 to 5.4 for improvement and 8.3 to 8.9 for worsening, using a patient global rating scale.

#### Chalder fatigue scale (CFS), score 0–33

The same two cross-sectional studies [33, 46] reported MIDs for the CFS where MIDs for global change were 2.3–3.3; for improvement 0.7–1.4; and for deterioration 3.2–3.5.

#### Fatigue impact scale (FIS), score 0–160

One cross-sectional study with MS patients [49] reported MIDs for the FIS ranging from 9–24 points for the different patient and clinician rating anchors, with a mean of 15.5 and SD 4.9. Distribution-based methods yielded MIDs ranging between 4.8–17.3 (1–2 SEM; ± 1/3-1/2 SD). Triangulation of anchor and distribution-based methods gave a MID range of 10–20 points.

#### Trial outcome index-fatigue (TOI-F), score 0–108

One study [28] reported TOI-F MIDs using data from three separate cancer trials. Triangulation was used to determine a MID, combining a patient-reported anchor, two physician-reported anchors (including response to treatment ratings), and one clinical anchor (haemoglobin level). MID estimates ranged from 4.8 to 26.6, and a single triangulated MID of 5.0 was recommended.

#### Perform questionnaire (PQ), score 12–60

One longitudinal study [22] estimated the PQ MID in cancer patients to be 3.7 for improvement. Triangulation was used to estimate a recommended MID of 3.5.

#### Schwartz cancer fatigue scale (SCFS), score 3–30

A longitudinal study of the SCFS using a patient-rated anchor [51] reported MIDs for global change was 5.0; for improvement 2.1; and for deterioration 5.7 after a two days follow-up.

#### Fatigue associated with depression questionnaire (FAsD), score 1–5

MIDs for the FAsD were estimated in one longitudinal study [43] of patients with a clinical diagnosis of depression ranging from 0.3 to 0.6 for improvement and 0.2–0.3 for worsening after 6 weeks follow-up.

#### Neurological fatigue index for multiple sclerosis (NFI-MS), summary score 0–30

One longitudinal study [44] using a patient global assessment of change reported MIDs for the NFI-MS; 2.5 for the ten-item Summary scale, 2.4 for the Physical scale (score range 0–24) and 0.8 for the Cognitive scale (score range 0–12).

### Unidimensional scales or subscales

#### Multidimensional fatigue inventory (MFI) subscales, score 4–20

A longitudinal study [47] derived MIDs in a cancer population (pre and post radiotherapy) for the MFI five subscales. MIDs ranged between 1.4 to 2.4 depending on subscale. A general MID for all MFI subscales was recommended corresponding to 2 points.

#### Unidimensional fatigue impact scale (U-FIS), score 0–66

One longitudinal study using EQ5D as an anchor [55] derived MIDs in an MS sample. U-FIS MIDs corresponded to 6.5 for improvement and 4.7 for deterioration, and distribution-based MIDs between 2.4 and 7.0.

#### Fatigue assessment scale (FAS), score 10–50

MIDs for the FAS were reported in one longitudinal study of sarcoidosis patients using WHOQOL-BREF/Physical health domain and a ROC-curve as anchors as well as distribution based methods [31]. MID ranged between 3.0 and 4.2 and a triangulated MID-value of 4 was suggested.

#### Vitality scale (VT) of the medical outcome study SF-36 health survey (SF-36), score 0–100

Eight studies [26, 33, 35, 46, 54, 56, 59, 60] determined MIDs for the VT scale of the SF-36 using different designs and diagnostic groups; longitudinal with patient- and/or clinician rated anchors, cross-sectional using patient-rated anchors and systematic reviews using combined study data and expert panels. The MIDs ranged from 7.3 to 11.3 for improvement, 11.9 to 18.3 for worsening and 3.5 to 20, for all those with a global change and 4.2 to 18.8 for a triangulated MID.

#### FACIT fatigue scale (FACIT-Fatigue), score 0–52

Six cross-sectional or longitudinal studies [28, 29, 33, 38, 46, 48] reported MID estimates derived in patients with cancer, SLE, or RA using patient or clinician-rated anchors. In these studies, MIDs varied from 3 to 8.3 irrespective of direction of change, 2.8 to 6.8 for improvement and 5.2 to 9.1 for deterioration. Two of the studies [29, 38] combined various distribution-based approaches (SEM, SD and ES), resulting in MIDs ranging between 2.2 and 6.8, and presented triangulated MIDs ranging between 3 and 6.

#### FACT-an fatigue subscale (FACT-An Fatigue), score 0–80

One longitudinal study [45] estimated a MID for improvement of 4.2 in cancer patients using haemoglobin level as a clinical anchor and regression analysis to calculate MID.

#### Profile of mood states short form fatigue subscale (POMS-F), score 0–28

One longitudinal study reported MIDs for the POMS-F using a sample of cancer patients undergoing chemotherapy [51]. A global MID of 5.6 points was determined as well as separate MIDs for improvement (2.1 points) and deterioration (5.7 points).

#### European organization for research and treatment of cancer quality of life questionnaire core 30 (EORTC QLQ-30)—fatigue scale, score 0–100

Six cross-sectional and longitudinal studies [24, 25, 36, 40, 41, 62] reported MIDs derived in a variety of cancer diagnoses. MIDs were reported as 11.4 to 17.3 points for improvement and 5.7–24.5 points for deterioration. Distribution-based MIDs ranged from 3.0 to 19.7.

#### Sleep impact scale (SIS), energy/fatigue and mental fatigue subscales, score 0–100

One longitudinal study [39] using a clinician-rated anchor and a distribution-based method to assess change at 8-week follow-up, reported MIDs derived in patients with major depressive disorder (MDD). The anchor-based approach yielded a MID of 11.9 for the Energy/Fatigue subscale, whereas the distribution-based MID was 8.7. The corresponding MIDs for the Mental Fatigue subscales were 13.3 and 10.6, respectively.

#### Chronic respiratory questionnaire (CRQ), score 1–7

Two systematic reviews [52, 59] used CRQ data from earlier studies to determine MIDs for the CRQ/Fatigue subscale and triangulated MIDs of 0.5 and 2 were proposed. One of the reviews estimated MIDs between 0.5–0.6 for global change and distribution-based MIDs of 0.47–0.54 [52].

#### Chronic heart failure questionnaire (CHQ), score 4–28

One systematic review using CHQ data and an expert panel proposed a MID for the CHQ/Fatigue subscale of 3–4 irrespective of direction and a triangulated MID of 3 [60].

#### Quality of life inventory in Epilepsy (QOLIE-31), energy/fatigue subscale, score 0–100

One longitudinal study used 3 randomised controlled trials to examine MID for the QOLIE-31/Energy/fatigue subscale [27]. A MID of 7.5 was defined using a patient rating of change and regression analysis. Distribution-based MIDs ranged between 5.4 and 9.4.

#### Visual analogue scale (VAS), score 0–100 or 0–10

Six longitudinal studies [30, 32, 37, 53, 57, 58] derived MIDs for the VAS 0–100 and one [34] for the VAS 0–10 in a variety of diagnostic groups. MIDs for the VAS-100 ranged from 1.4 to 13.9 for improvement and 3.6 to 15.2 for deterioration, while the global change varied between 6.7 and 17. One study [57] determined a triangulated MID of 10 using the Delphi method. MIDs for the VAS-10 ranged between 0.8 to 1.1 for improvement and 1.1 to 1.3 for worsening, and were derived from three different anchors and at different follow-up times in three different diagnostic groups (RA, SLE and cancer) [34].

#### Global rating scale (GRS), score 0–10

MIDs for the single item GRS scale were determined in SLE, RA and cancer patients in two cross-sectional studies [33, 46] and one longitudinal study [51], all using a patient global rating scale as an anchor. Global MIDs ranged from 1.1 to 2.0, while MIDs for improvement were 0.3 to 0.9 and for deterioration 1.5.

#### Edmonton symptom assessment system (ESAS) fatigue item, score 0–10

Two longitudinal cancer studies [23, 48] identified MIDs for the fatigue item in the ESAS scale. MIDs for improvement ranged from 0.1 to 4 and between 1.0 and 1.8 for worsening of fatigue. Distribution-based MIDs ranged from 0.1 to 1.4.

#### Immune thrombocytopenic Purpura—Patient assessment questionnaire, (ITP-PAC) fatigue subscale, score 0–100

One longitudinal study [42] assessed MIDs using patient impression of change for the ITP-PAC/Fatigue subscale. Global change was defined as 15.0 or as an effect size of 0.57.

### PROMIS fatigue item bank scales

#### 17-item PROMIS fatigue (fatigue-17) and 7-item PROMIS Fatigue (Fatigue-7), score 17–85 and 7–35

One study [61] derived MIDs for both the PROMIS Fatigue-17 and Fatigue-7 in patients with cancer. The study used both cross-sectional and longitudinal data as well as anchor-based and distribution-based methods. Distribution-based MIDs were reported as effect sizes. For the Fatigue-17, the ES ranged from 0.34–0.79 and 0.27–0.52 for cross-sectional and longitudinal designs, respectively. Corresponding effect sizes for the *Fatigue-7 were* 0.24–0.76 and 0.24–0.51. Triangulated raw score MIDs ranged from 4.0 to 8.0 for the Fatigue-17 and 2.0 to 3.0 for the Fatigue-7 while t-score MIDs varied between 2.5 to 4.5 for the Fatigue-17 and 3.0 to 5.0 for the Fatigue-7.

## Discussion

This systematic review identified 41 studies reporting MIDs for 28 fatigue PROMs or subscales measuring fatigue, yielding a total of 60 studies or substudies estimating MID. It is important to note that there are many more fatigue PROMs available today than the 28 reported here. For example, a critical review of fatigue PROMs from 2009 [8] identified 39 such PROMs; however, only 11 of these overlapped with PROMs in our review. This suggests that there are roughly 56 or more fatigue PROMs currently represented in the literature. Considering the importance attributed to MIDs for interpreting the meaningfulness of change in PROM scores [9, 63], it is somewhat surprising that MIDs are available for only about half of all published fatigue PROMs. Moreover, few PROMs had MIDs that were determined in more than two studies and diagnostic groups, and more than half of the PROMs had MIDs that were derived in only one diagnostic group. Important exceptions were the SF-36 Vitality scale (>8 diagnostic groups/8 studies; the FACIT-Fatigue scale (4 diagnoses/6 studies); the EORTC QLQ-C30 Fatigue subscale (6 cancer diagnoses/6 studies); and the VAS-100 Fatigue (6 diagnoses/6 studies). Considering that these scales are some of the most widely used and oldest PROMs in use today it is unsurprising that greater research attention has focused on determining MIDs for these scales; however, it is noteworthy that so few separate studies reported MIDs for commonly used generic fatigue PROMs, such as the MFI, FSS, FIS and FAS.

Previous research has highlighted considerable variability in MID values as a function of estimation method, population and context [14, 18, 19], suggesting the importance of considering such factors when appraising the appropriateness of published MIDs for use in clinical research and practice. In line with this, substantial variation was observed in MID values for individual fatigue PROMs in this review. For example, MIDs for the SF-36 Fatigue scale ranged from as low as 4.2 to as high as 20.0 points (0–100 point scale) in studies varying in methodologies, anchors, diagnostic groups and direction of change assessed. Similarly, MIDs for the VAS-100 Fatigue scale ranged from 1.4 to 17. MIDs for the cancer-specific EORTC QLQ-C30 fatigue scale also varied between 1.8 and 24.5 points (0–100 scale) and those for the FACIT-Fatigue scale ranged between 6 and 16 (converted to percent), see Table 3. This wide variation in MIDs for individual fatigue scales suggests the importance of understanding how any particular MID was derived and of applying this knowledge when appraising its appropriateness for interpreting changes in fatigue scores.

MID estimation methods varied considerably in the identified studies and substudies. However, in accordance with recent recommendations regarding methods for MID estimation [14], nearly all studies applied an anchor-based approach, where at least one anchor was used. Patient global change ratings were by far the most common anchor, but even clinician-reported and clinical anchors were implemented. Where more than one anchor was applied either a range of values was generally reported or, as recommended [14, 63, 64], values were often triangulated to a single or smaller range of MIDs. Distribution-based methods were used in about a third of the studies and only in conjunction with anchor-based approaches. A few studies used a Delphi method (Table 3).

In the studies using several anchors to determine MID values, global MID ranges varied within single studies from as little as two points (percent scores), in relation to the FACIT-Fatigue scale using patient-based anchors [29], to about 20 points for the TOI-F [28] using patient, clinician and clinical anchors. Interestingly, two studies reporting MIDs for the SF-36 Vitality scale, using the same diagnostic group (RA) but different anchors, yielded two distinct ranges of MIDs. In the study by Kosinski et al. [35], using patient and physician global assessments as anchors, MIDs ranged from 4.9–11.1, whereas a range of 11.0–20.0 was reported by Ward et al. [56] using the HAQ, CES-D and the SF-36 health transition item. Neither of these studies triangulated the range of values to a single MID or smaller range of values and hence these wide ranges of MIDs are arguably of questionable practical value for interpreting change in fatigue in RA patients as measured with the SF-36 Vitality.

Triangulation was used in 17 substudies, of which 10 used more than two anchors. This method has been recommended for consolidating MIDs derived from different methods to a single or small range of MID values [14]. However, it has been criticized [19] since it may in practice involve the need to converge widely disparate MIDs derived using different estimation methods and diverse anchors, which often represent very different stakeholder perspectives. An example of a MID triangulated from a wide range of MIDs is the TOI-F [28] where a MID range of 4.4–24.6 (percent scores) was triangulated to 5.0. Where MID ranges are smaller, the value and applicability of the triangulated MID may be more immediately apparent. For example, Schünemann et al. [52] reported a MID range for the CRQ of 6.7–8.5 (percent scores), derived from patient anchors, a systematic review and distribution-based methods, which was triangulated to a MID of 6.7.

A second factor known to influence variation in MID values is the patient population in which the MID is determined. Variation by diagnostic group is exemplified by comparing MIDS from two studies, each using the same estimation method (7-step global rating scale) and study design (cross-sectional) but different diagnostic groups [33, 46]. One of the studies [33] determined MIDs for seven different fatigue PROMs in patients with SLE and the other [46] did the same in patients with RA. Comparison of the global MIDs for the SLE and RA patients, shown in Table 3, shows consistently smaller MIDs for SLE versus RA across all seven PROMs. It is noteworthy that most PROMs had MIDs that were determined in only one patient population and the relevance of these MIDs for use in other patient groups thus remains unclear.

A third factor influencing variation in MID values is the context within which the MID is determined. Context issues concern, for example, characteristics of the patient population, e.g., such as baseline state [65], disease severity [66], and direction of change [13, 20], as well as study design and intervention. For example, patients with baseline scores indicating more severe fatigue may value magnitudes of change in fatigue differently than those with less severe fatigue. Corroborating previous research finding, MIDs for improvement differed from those for deterioration in all identified studies. MIDs tended to be larger for deterioration than improvement, except in the EORTC QLQ-30 and VAS Fatigue item. MIDs for improvement were consistently smaller than global MIDs.

A strength of this study is that reported MIDs for fatigue scales or subscales were systematically compiled and described. Assessment for inclusion or exclusion and data extraction from included studies was done independently by two authors (ÅN and AD). A limitation is that the search period was restricted to studies from 2000 onwards and search strings for the many variations on MID was also limited and therefore some studies reporting MIDs for fatigue scales may not have been captured in the literature searches. Another limitation is that the description of the study designs and results had to be summarized and simplified in tables and information could be lost. Therefore, when evaluating MIDs the original study/studies should be consulted.

## Conclusions

MIDs vary substantially by estimation method, patient population and context both across and within fatigue PROMs. In light of this variation, published MIDs should be applied judiciously, after carefully considering their applicability to characteristics of the study in question. The information provided in this paper may serve to aid researchers and clinicians in making informed decisions regarding the appropriateness of published MIDs for their particular study and patients.

## Abbreviations

ES, effect size; GRS, global rating scale; ITP, immune thrombocytopenic purpura; MCID, minimal clinical important difference; MCII, minimal clinically important improvement; MDD, major depressive disorder; MID, minimal important difference; MS, multiple sclerosis; PRO, patient reported outcome; PROM, patient reported outcome measure; PROMIS, patient-reported outcomes measurement information system; RA, rheumatoid arthritis; SD, standard deviation; SEM, standard error of measurement; SLE, systemic lupus erythematosus; QoL, quality of life.
